# Matched Metabolic Stress Preserves Myokine Responses Regardless of Mechanical Load: A Randomized, Controlled Crossover Trial

**DOI:** 10.3390/metabo15100641

**Published:** 2025-09-25

**Authors:** Yuji Maki, Hiroo Matsuse, Ryuki Hashida, Norika Matsukuma, Hiroshi Tajima, Eriko Baba, Yuji Kaneyuki, Sohei Iwanaga, Masayuki Omoto, Yoshio Takano, Matsuo Shigeaki, Takeshi Nago, Koji Hiraoka

**Affiliations:** 1 Rehabilitation Center, Kurume University, 67 Asahi-cho, Kurume 830-0011, Fukuoka, Japan; maki_yuuji@med.kurume-u.ac.jp (Y.M.); hashida_ryuuki@med.kurume-u.ac.jp (R.H.);; 2 Department of Physical Therapy, School of Health Sciences at Fukuoka, International University Health and Welfare, 137-1 Enokizu, Okawa 831-8501, Fukuoka, Japan; 3 Department of Mechanical Systems Engineering, Faculty of Engineering, Kurume Institute of Technology, Graduate School of Energy System Engineering, 2228-66 Kamitsu-machi, Kurume 830-0052, Fukuoka, Japan

**Keywords:** skeletal muscle, myokine, mechanical stress

## Abstract

**Background/Objectives**: Skeletal muscle functions as an endocrine organ by secreting myokines in response to exercise, with interleukin-6 (IL-6) recognized as a representative intensity-dependent biomarker that rapidly increases immediately after exercise and is strongly dependent on exercise intensity. However, it is unclear how changes in mechanical stress affect the response of myokines after exercise. This randomized crossover study aimed to investigate the effect of mechanical stress on acute myokine secretion during matched metabolic exercise under different mechanical stress. **Methods**: Ten healthy adult males performed 30 min of cycling at 60% of peak $\overset{\cdot }{V}$O_2_ in both semi-recumbent position and side-lying positions. Blood samples were collected before, immediately after, and at 30 and 60 min post-exercise to evaluate IL-6, brain-derived neurotrophic factor (BDNF), and lactate. **Results**: BDNF and lactate levels peaked immediately after exercise, and IL-6 reached its peak at 30 min post-exercise in both the semi-recumbent position and side-lying positions. All markers showed significant elevations in response to exercise. However, no significant differences were found between the two postures in any of the measured variables. **Conclusions**: These findings suggest that reduced mechanical load does not impair endocrine responses when the intensity of metabolic stress is maintained. This study provides scientific evidence that, regardless of posture or environment, sufficient exercise intensity can induce adequate IL-6 and BDNF secretion, through which the beneficial effects of exercise may be expected.

## 1. Introduction

Recent research has shown that skeletal muscle functions not only as a motor organ but also as a significant endocrine regulator, contributing to whole-body homeostasis as the largest endocrine organ in humans. Myokines—secreted factors from skeletal muscle—have gained attention for their role in metabolic regulation, immune modulation, and inter-organ communication. The discovery of interleukin-6 (IL-6) by Pedersen et al. marked the beginning of this research, with IL-6 secreted from skeletal muscles during exercise being implicated in anti-inflammatory effects and improvements in glucose and lipid metabolism [1]. Following the discovery of IL-6’s role, brain-derived neurotrophic factor (BDNF) has also emerged as a key myokine with distinct metabolic and neurological effects. Furthermore, brain-derived neurotrophic factor (BDNF) promotes lipid oxidation through AMPK activation within skeletal muscle, contributing to improved energy metabolism efficiency, enhanced endurance, and prevention of metabolic diseases. In the central nervous system, BDNF contributes to neurogenesis and cognitive function improvement [2]. Thus, myokines exert a wide-ranging influence across multiple organs. However, there is no consensus on the differences in myokine secretion between mechanical and metabolic stimuli [3,4,5]. Mechanical and metabolic stimuli have been proposed as the primary factors regulating myokine secretion [1,6]. Mechanical stimulation results from tension and strain on muscle fibers and the extracellular matrix, typically occurring during high-load resistance or eccentric exercise. On the other hand, metabolic stimulation is induced by the accumulation of metabolic byproducts such as lactate and H^+^ ions during moderate-to-high intensity aerobic exercise at above the anaerobic threshold (AT).

A side-lying ergometer (SLE) is an experimental model that has garnered attention for its ability to selectively control mechanical stimuli. This device supports subjects in a side-lying or supine position and can reduce weight-bearing load by partially or completely unloading body weight. As a result, it has been utilized as a ground-based method for replicating microgravity environments, and similar devices such as NASA’s Zero Gravity Locomotion Simulator have been widely used to mimic physiological changes under microgravity conditions [7,8,9]. Furthermore, it has been reported that in microgravity environment, bone resorption and bone formation become dissociated, and myokines play an important role as regulatory factors [10]. By examining myokine secretion responses to exercises with different mechanical stresses, it is possible to determine whether exercise performed in restricted environments, such as in various postures or in limited spaces, can still elicit beneficial effects mediated by myokines. Therefore, this study standardizes metabolic stress based on a heart rate equivalent to 60% of peak $\overset{\cdot }{V}$O_2_, conducts exercise tests under different loading conditions (semi-recumbent position and side-lying position), and verifies the secretion dynamics of IL-6 and BDNF as well as changes in blood lactate concentration.

## 2. Method

### 2.1. Definitions of Terms

Side-lying positions—defined as postures in which the participant lies on one side of the body, either the left or the right.

Side-lying ergometer—defined as exercise performed in a sideways position while pedaling an ergometer.

Semi-recumbent positions—defined as a posture in which the participant lies in a half-reclined position with the upper body elevated, typically at an angle of 30–45 degrees.

Semi-recumbent ergometer—defined as exercise performed in a semi-recumbent position while pedaling an ergometer.

### 2.2. Participants

Twelve healthy young men agreed to participate (Table 1).

All participants were provided with an explanation of this study’s purpose and methods, both verbally and in writing, and written consent to participate in this study was obtained, in accordance with the Declaration of Helsinki. Participants were required to meet all of the following inclusion criteria: (1) aged 20 to 45 years, (2) non-smokers, and (3) no regular exercise habits within the past six months. Physical function was evaluated by orthopedic and rehabilitation specialists based on physical findings such as muscle strength, sensation, and joint range of motion, in accordance with the criteria established by the Japanese Orthopedics Association. Exclusion criteria included the following: (1) history of musculoskeletal disorders, (2) history of conditions affecting cardiopulmonary function, and (3) currently receiving medical treatment. This study aimed to accumulate fundamental data, and therefore only male participants were recruited to minimize physiological confounding factors such as hormonal fluctuations and the potential influence of pregnancy. These criteria were established to minimize confounding factors that could influence the interpretation of exercise tests and myokine responses in this study. During the study period, participants were instructed to refrain from regular sports activities.

### 2.3. Study Design

**Figure 1:**
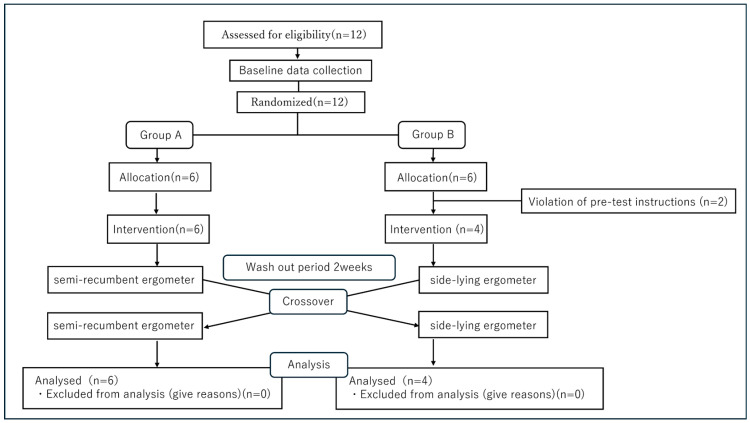
A flow chart in this study.

This experiment was conducted as a preliminary study. The flowchart of this study is presented in Figure 1. This randomized crossover trial was conducted in the clinical laboratory of a university-affiliated rehabilitation center. Participants performed aerobic exercise under two different postural conditions. SRE refers to exercise performed on a recumbent ergometer in the semi-recumbent position. SLE refers to exercise performed on a recumbent ergometer in the side-lying position. In addition, a two-week washout period was implemented between exercise sessions to eliminate any carryover effects.

Twelve subjects were screened, and randomization was performed on the day of the initial intervention. Two subjects assigned to Group B were excluded due to non-compliance with pre-test restrictions. Consequently, Group A comprises 6 subjects and Group B comprises 4 subjects. During Period 1, Group A performed semi-recumbent ergometer exercise, while Group B performed lateral recumbent exercise. After a 2-week washout period, the groups crossed over. All 10 subjects completed both periods, and their data was included in the final analysis.

The rationale for employing this design was twofold: (1) it was not feasible to completely blind participants and investigators to the order of exercise conditions, and (2) it was necessary to minimize the impact of inter-individual variability. The crossover design ensured that each participant performed both conditions, thereby controlling for potential confounding between subjects. All exercise sessions were conducted on the same day of the week and at the same time for each subject, under controlled conditions with a temperature of 21–24 °C and a relative humidity of 45–55%.

### 2.4. Base Data Collection

The following procedures and conditions were applied uniformly to all subjects. First, cardiopulmonary exercise testing (CPX) using the ramp load test was performed to set the exercise intensity for each subject during aerobic exercise. CPX was performed using the ramp load method, and the peak oxygen consumption (Peak $\overset{\cdot }{V}$O_2_) of each subject was calculated. Based on these results, the exercise intensity during aerobic exercise was set to a heart rate equivalent to 60% of Peak $\overset{\cdot }{V}$O_2_, exceeding AT and the exercise load was adjusted to ensure that the target heart rate was always exceeded during each exercise test.

All subjects underwent CPX using a ramp exercise test 2–4 weeks before the first intervention.

### 2.5. Intervention

Participants were instructed to refrain from alcohol and caffeine intake for 48 h prior to the exercise test. To minimize the influence of diurnal variations on metabolic and hormonal responses, all tests were uniformly started at 9 AM. In addition, breakfast on the day of the test was standardized to approximately 500 kcal with identical content for all participants. Subsequently, baseline blood sampling was performed before both the RE and SLE tests in participants who strictly adhered to these protocols (details are described later). After baseline blood sampling, a 30 min rest period was provided, after which participants performed either SRE or SLE for 30 min. Post-exercise blood samples were collected at three time points: immediately after exercise, 30 min after exercise, and 60 min after exercise.

### 2.6. SRE Test Protocol

In the SRE condition, the exercise load was set so that each subject reached the target heart rate (equivalent to 60% of Peak $\overset{\cdot }{V}$O_2_) within 3 min of exercise onset, after which aerobic exercise was initiated. During exercise, HR was monitored using a pulse oximeter, and adjustments were made to maintain the target heart rate. Audio encouragement was also used to help maintain heart rate. The exercise load was set at a constant load of 60% of Peak $\overset{\cdot }{V}$O_2_, and continuous exercise was performed for 30 min. Pedaling cadence was instructed to be maintained at 60–80 revolutions per minute (rpm) using a pedal revolution counter.

We used the SportsArt medical recumbent bike (model CM521, SportsArt, headquarters; Mukilteo, Washington, United States), whose workload control system was based on an electromagnetic brake under the SRE condition.

### 2.7. SLE Test Protocol

**Figure 2:**
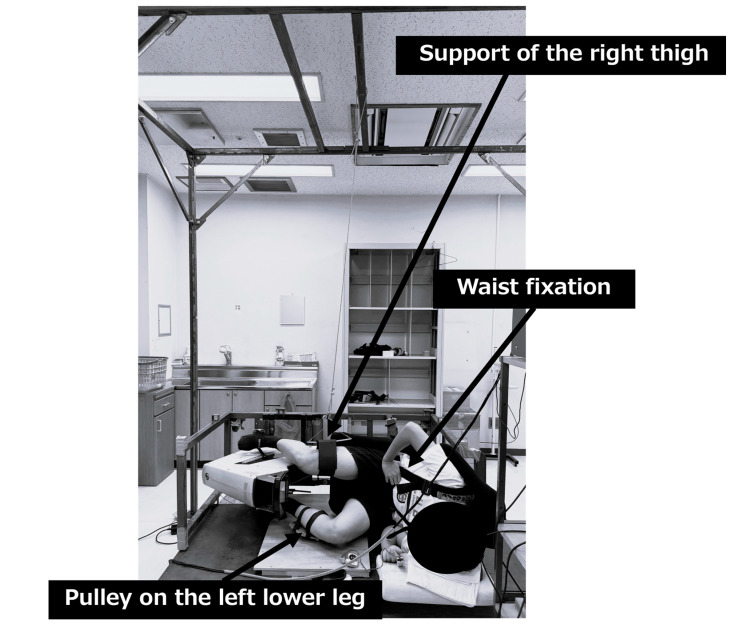
Setup for the supine lateral exercise condition.

The SLE condition was conducted under the same exercise conditions with the same exercise stress and duration as the SRE condition. However, since the posture differed in that the subjects exercised in lateral position, the following additional conditions were set. During exercise, the subjects secured their waist with a seat belt to stabilize the trunk. The right thigh was suspended from the ceiling and supported so that it was parallel to the ground. A pulley was attached to the left lower leg and adjusted to allow smooth pedaling in a direction parallel to the ground. The exercise posture was standardized to the left side-lying recumbent position for all subjects (see Figure 2).

We used the SLE apparatus, constructed in our laboratory on the basis of a Cateye Ergociser EC-3600, and its workload control system was based on an electromagnetic disk brake, ensuring that the applied resistance was unaffected by gravity even when the apparatus was positioned horizontally.

The subject performed the exercise in the left lateral position. The torso was secured with a seatbelt (lumbar fixation), the right thigh was suspended parallel to the floor (right thigh support), and a pulley was attached to the left lower leg to maintain pedaling parallel to the floor (left lower leg pulley). Exercise load was applied as pedal resistance (kgm). In this study, exercise load intensity was adjusted based on heart rate. The adjustment device was jointly developed by our laboratory based on the Cateye Ergociser EC-3600.

Labels: (1) Lumbar fixation belt; (2) right thigh suspension support; (3) left lower leg pulley system.

### 2.8. Blood Sampling

**Figure 3:**
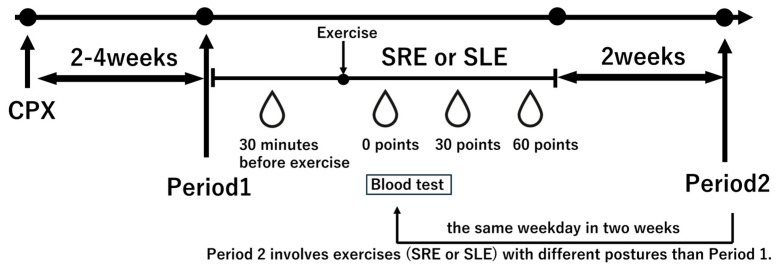
Study protocol. Subjects underwent a cardiopulmonary exercise test (CPX) 2–4 weeks prior to the experimental intervention to determine the exercise load. In Period 1, subjects performed the randomly assigned exercise, either semi-recumbent ergometer (SRE) or side-lying ergometer (SLE), followed by a 2-week washout period. In Period 2, they performed the alternate exercise on the same weekday as in Period 1. All subjects consumed a standardized 500 kcal breakfast at 7 AM, and the experiment began at 9 AM. Blood samples were collected 30 min before exercise, immediately after exercise, 30 min post-exercise, and 60 min post-exercise. SRE: Semi-Recumbent Ergometer; SLE: Side-Lying Ergometer.

Prior to the session, subjects rested in a seated position for 30 min, after which baseline blood samples were collected. Blood samples were also collected immediately after exercise (0 min), as well as 30 and 60 min post-exercise (see Figure 3).

At each measurement point, 20 milliliters of blood were collected via a cannula inserted into the elbow vein while the subject was in a supine position. All blood samples were centrifuged for 10 min, and the resulting plasma was stored at −20 °C until analysis. Serum sampling analysis was performed by SRL (S.R.L. Co., Ltd., Tokyo, Japan). The concentration of BDNF (brain-derived neurotrophic factor) in plasma was measured using an enzyme-linked immunosorbent assay. IL-6 in plasma was measured using a chemiluminescent immunoassay. Lactate concentrations in plasma were measured using the lactate oxidase method and an automatic analyzer (JCA-BM8000; Japan Electron Optics Laboratory, Tokyo, Japan) (Determiner LA; Kyowa Medex Co., Ltd., 1-8-10 Harumi, Chuo-ku, Tokyo).

### 2.9. Statistical Analysis

The normality of all data was tested. For normally distributed data, paired *t*-tests were used, and for non-normally distributed data, Wilcoxon signed-rank tests were applied to compare pre- to post-exercise changes in both exercise conditions, with Bonferroni correction for multiple comparisons. Furthermore, to compare differences between exercises, a crossover analysis was conducted with Treatment (exercise intervention), Period, and Sequence as fixed effects, and subjects (nested within Sequence) as random effects to examine carryover effects, period effects, and sequence effects. Additionally, we conducted further analyses on the primary results. Statistical analyses were performed using JMP 11.0 (JMP Statistical Discovery LLC, 920 SAS Campus Drive Cary, NC 27513.), with the significance level set at *p* < 0.05.

In addition, the effect sizes (Cohen’s *dz*) were calculated after the intervention under each condition.

### 2.10. Sample Size Calculation

The sample size of the present study was calculated based on the report by Cabral-Santos et al. (2016) [11]. In their study, healthy young men performed high-intensity intermittent exercise, and changes in inflammatory cytokines, particularly IL-6, were evaluated before and after exercise. IL-6 showed a significant increase immediately after exercise, and the effect size was derived from the pre–post change data [11]. In this study, statistical power was set at 0.8 and the α error at 0.05. Assuming an effect size of 0.91 and accounting for a 15% dropout rate, the required sample size was calculated. As a result, calculations using G*Power version 3.1.9.7 indicated that nine participants were required. Considering a safety margin, we set to recruit 12 subjects for this study.

## 3. Results

A total of 12 subjects underwent eligibility assessment. As shown in Figure 1, subjects were randomly assigned to two sequences (Group A: SRE→SLE/Group B: SLE→SRE). During the pre-intervention interview, two participants were identified as unable to comply with the alcohol and caffeine restriction 48 h prior to the exercise test and were excluded before the exercise intervention. Therefore, no intervention data was obtained for these excluded subjects. After exclusion, Group A consisted of 6 subjects and Group B consisted of 4 subjects. All subjects completed the trial. No difficulties in continuing due to pain or fatigue, nor any adverse events, were observed during the exercise stress test.

Baseline information collected at screening for the analysis group (*n* = 10) and the excluded participants (*n* = 2) is presented in Supplementary Table S1. Standardized mean differences (SMDs) between the analysis and excluded groups were 0.83 for age, 0.66 for BMI, 0.38 for height, 0.37 for body mass, and 0.43 for peak $\overset{\cdot }{V}$O_2_. Although some of these values indicate small-to-large differences, the excluded group comprised two participants who were removed after randomization but before the exercise intervention. The final sample size was 10 subjects (mean ± standard deviation [SD]: age 21.2 ± 0.9 years; height 173.8 ± 5.9 cm; weight 64.9 ± 8.3 kg; BMI 21.3 ± 2.0 kg/m^2^; peak $\overset{\cdot }{V}$O_2_ 33.2 ± 3.9 mL/kg/min).

In the crossover analysis using a fixed-effects model, no significant differences were observed for the primary outcomes of IL-6 or BDNF. By contrast, for lactate, the sequence effect was significant (estimate 17.22, 95% CI 2.87–31.57, *p* = 0.024) and the period effect was also significant (estimate 36.34, 95% CI 1.00–71.68, *p* = 0.045). The treatment (exercise posture) effect was not significant (between-posture difference −3.73, 95% CI −19.36 to 11.90, *p* = 0.599), and a borderline carryover effect was observed (estimate −35.72, 95% CI −72.94 to 1.42, *p* = 0.058) (Supplementary Table S2).

Based on these findings, we conducted additional exploration analyses. Residual diagnostics indicated centered residuals with approximately constant variance; however, the normal Q–Q plot showed slight tail deviations and a few outliers. In a parallel-group comparison restricted to period 1 (baseline lactate), values were 13.08 ± 2.57 in the SRE group and 10.10 ± 3.00 in the SLE group, with no statistically significant difference (Wilcoxon rank-sum test: two-sided *p* = 0.166; one-sided *p* = 0.083).

Taken together, these results suggest that lactate responses may be broadly comparable across exercise postures, while interpretation of treatment effects should consider sequence, period, and potential carryover effects.

In the time-series analysis, lactate increased significantly immediately after exercise under both conditions (SRE, *p* = 0.0072; SLE, *p* = 0.0009) (see Table 2).

IL-6 also increased significantly by 30 min after exercise under both conditions (SRE, *p* = 0.0132; SLE, *p* = 0.0021) (see Table 3).

In addition, BDNF increased significantly immediately after exercise in both conditions (SRE, *p* = 0.0024; SLE, *p* = 0.024) (see Table 4).

In addition, large effect sizes appeared to be observed under the RE condition for Lactate (*dz* = 1.32, 95% CI [0.32–2.32]), BDNF (*dz* = 1.56, 95% CI [0.46–2.66]), and IL-6 (*dz* = 1.19, 95% CI [0.24–2.15]). Similarly, under the SLE condition, large effect sizes were also observed for Lactate (*dz* = 1.79, 95% CI [0.60–2.99]), BDNF (*dz* = 1.07, 95% CI [0.16–1.99]), and IL-6 (*dz* = 1.59, 95% CI [0.48–2.70]).

## 4. Discussion

### 4.1. Summary of Main Results

In this study, we compared two conditions with different mechanical stress induced by gravity, semi-recumbent ergometer (SRE) and side-lying ergometer (SLE), in healthy individuals after adjusting the metabolic intensity to 60% of peak $\overset{\cdot }{V}$O_2_. Specifically, despite different mechanical load conditions, IL-6 showed a significant increase 30 min after exercise, while BDNF and lactate showed significant increases immediately after exercise. As a result, no significant differences were observed in IL-6, BDNF, or lactate levels between SRE and SLE conditions. The present findings may indicate that both IL-6 and BDNF could exhibit appropriate endocrine responses to exercise, regardless of postural conditions.

Previous studies have reported that serum IL-6 levels increase immediately after moderate-to-high intensity aerobic exercise, while serum BDNF levels also rise under the same conditions [11,12,13,14].

In this study, the observed changes in BDNF and IL-6 were generally consistent with these previous findings, which may support the notion that appropriate neurotrophic and inflammatory responses were elicited. This appears to align with the review findings by Curovic et al. that “local metabolic stress drives myokine responses,” lending support to the possibility that metabolic stimulation is a more important trigger than mechanical stress [15]. Therefore, sufficient physiological effects could be achieved through the deliberate modulation of metabolic stress, even in environments with restricted mechanical loading, such as in patients with physical and environmental limitations or in space environments. This approach may be applicable as an effective rehabilitation approach for elderly or immobile patients to maintain muscle function and metabolic health.

### 4.2. Characteristics of Myokine (IL-6, BDNF) Secretion Responses According to Aerobic Exercise Intensity

IL-6 is known to be promoted through glycogen depletion and Ca^2+^ signaling in skeletal muscle, and these responses depend on exercise intensity [16]. Furthermore, an increase in lactate, a metabolic indicator, has also been suggested to be associated with IL-6 secretion. Omoto et al. conducted a 40% of peak $\overset{\cdot }{V}$O_2_ cycle ergometer exercise on healthy adults and examined its effects on IL-6 secretion [17]. The results showed no significant changes in either lactate or IL-6 after exercise. This suggests that 40% of peak $\overset{\cdot }{V}$O_2_ exercise may be insufficient to induce metabolic stress and may be weak to stimulate IL-6 secretion. On the other hand, Abbotts KSS et al. reported that in healthy adults, 30 min of moderate-intensity ergometer exercise at 60% of peak $\overset{\cdot }{V}$O_2_ resulted in significant increases in lactate immediately after exercise and in IL-6 30 min after exercise [18]. In this study, similarly, both SRE and SLE conditions performed at 60% of peak $\overset{\cdot }{V}$O_2_ exercise intensity showed significant increases in IL-6 at 30 min post-exercise. Additionally, the lactate levels observed in this study exceeded the general lactate threshold of 2 mmol/L [19], suggesting that sufficient metabolic stress was induced. These results lend support to the possibility that the IL-6 secretion response is primarily caused by metabolic stress.

In this study, BDNF increased under both the resistance exercise and single-leg exercise conditions after exercise, and an increase in lactate levels was observed in both conditions. These results are consistent with previous studies reporting that lactate increase and BDNF increase occur in parallel [20]. Furthermore, Schiffer et al. demonstrated that lactate administration to young adults at rest significantly increased BDNF. Furthermore, a meta-analysis on BDNF and exercise intensity reported that moderate-to-high-intensity exercise significantly promotes BDNF secretion, and that the increase in lactate levels, as an indicator of exercise intensity, is associated with an increase in BDNF concentration [21]. Considering these findings, it is possible that Metabolic stress may play an important role in promoting BDNF secretion after exercise. However, other physiological factors may also be involved in BDNF secretion after exercise, and further investigation is needed to clarify the underlying mechanisms.

### 4.3. Bone and Muscle Adaptation and Myokine Responses in a Low-Gravity Environment

In this study, we confirmed that myokine secretion, such as IL-6 and BDNF, is maintained even in a low-mechanical load environment using the SLE with moderate intensity. This result is an important finding suggesting that IL-6 and BDNF responses depend not only on the presence or absence of weight-bearing stimuli but also on metabolic load itself. IL-6 and BDNF are skeletal muscle-derived myokines known to contribute to inflammation control, metabolic regulation, and promotion of neuroplasticity [22,23] and maintaining their secretion is directly linked to overall health maintenance.

Previous studies have shown that IL-6 secreted from muscles during exercise functions as a metabolic regulatory myokine [24] and has been reported to be associated with mental health [25]. In addition, BDNF, which increases with exercise, is involved in the alleviation of depressive symptoms and the promotion of neuroplasticity and has been shown to contribute to improvements in both physical and mental health [26]. Thus, IL-6 and BDNF secreted after exercise are mutually related in that they both contribute to physical and mental health.

On the other hand, in clinical practice, exercise environments for older adults and bedridden patients are often limited to the bed or bedside, where resistance exercise is frequently employed. However, under these conditions, it is difficult to obtain sufficient mechanical stress derived from gravity. The present findings suggest that adequate metabolic stress may help maintain appropriate myokine secretion through exercise and could potentially contribute to health, which may have relevance from a clinical application perspective.

Additionally, the findings of this study could be applied as an effective method for maintaining exercise under space-saving and low-load conditions in special environments such as spaceflight or lunar bases. In outer space, mechanical stress is markedly reduced. These findings suggest that, even under postural or environmental conditions with restricted mechanical stress, adequate metabolic stress may still induce sufficient myokine secretion through exercise. However, weight-bearing stimulation is essential for maintaining the structure of bones and muscles. In space or weightless environments, where there is no gravitational load, muscle atrophy and bone loss have been repeatedly reported [27,28]. In other words, it would be necessary to distinguish between maintaining metabolic responses and maintaining the structural integrity of bones and muscles. This duality, that metabolic responses can be maintained but mechanical stress is necessary for structural maintenance, represents an extremely important perspective when designing effective exercise strategies in low-gravity environments or clinical settings. However, further research is needed to elucidate this concept. In addition, it remains unclear whether the postural influence observed here is specific to cycling or is consistent across other exercise modalities. Future studies should therefore examine different types of exercise, including running and resistance training. Moreover, this study did not establish links between endocrine responses and real-world health benefits. Relating changes in IL-6 and BDNF to functional outcomes such as cognition, metabolism, and muscle performance will be essential to clarify their clinical significance.

### 4.4. Limitations

This study has several limitations. First, the sample comprised 10 healthy young men, which restricts external validity; future studies including women, older adults, and clinical populations are needed to improve generalizability. Second, we assessed only acute responses; thus, long-term training effects and the presence of physiological adaptation remain unclear. Third, the side-lying ergometer was used as a surrogate for microgravity but does not fully replicate true weightlessness. In addition, cycling was the sole exercise modality, which may limit generalizability to other activities such as running, resistance training, or functional tasks with different loading patterns. Moreover, mechanotransduction signaling (e.g., FAK and the YAP/TAZ pathway) was not measured, warranting further work to elucidate local molecular mechanisms. Although metabolic stress appeared comparable between conditions, we cannot confirm that all exercise parameters other than posture were identical. Fourth, two participants were excluded after randomization but before the intervention, raising the possibility of selection bias. The excluded participants differed from the analyzed cohort at baseline, potentially limiting representativeness and external validity. Although the small number of exclusions likely limited their influence on point estimates, post-randomization exclusions precluded a strict intention-to-treat analysis and may bias treatment effect estimates if the exclusions were related to assignment or outcomes. Future studies should pre-register exclusion criteria, report reasons in detail, and incorporate sensitivity analyses to quantify and mitigate selection bias. Fifth, several statistical considerations apply to our crossover analysis: (i) significant sequence and period effects and a borderline carryover effect suggest that time trends and residual effects from the preceding period may have influenced the estimated treatment effect; (ii) residual diagnostics showed slight tail deviations and a few outliers, indicating that the assumptions of normality and homoscedasticity were only approximately met—accordingly, estimates may be sensitive to distributional assumptions, and robustness/sensitivity analyses are warranted; and (iii) the additional analyses were exploratory and no formal multiplicity adjustment was applied, so these findings should be interpreted as hypothesis-generating. Taken together, these considerations suggest that SRE and SLE may elicit acute physiological responses of broadly comparable magnitude, but this interpretation should be made with caution.

Despite these limitations, we believe that the present findings provide meaningful insights into the acute cardiovascular and endocrine responses to exercise under simulated microgravity conditions, and they may serve as a valuable basis for future research.

## 5. Conclusions

Our findings suggest that maintaining a constant metabolic stress may preserve the endocrine responses of IL-6 and BDNF, regardless of mechanical stress such as posture or loading conditions. This suggests that appropriate metabolic regulation may compensate for differences in mechanical stress, providing important implications for exercise prescription in both clinical and low-gravity environments.

## Figures and Tables

**Table 1:** Baseline characteristics of participants.

Characteristic	Value (*n* = 12)
Age (year)	21.3 ± 0.9
Sex (male/female)	male (100%)
Height (cm)	174.1 ± 5.5
Weight (kg)	64.4 ± 7.6
BMI (kg/m^2^)	21.1 ± 2.0
Peak $\overset{\cdot }{V}$O_2_ (mL/kg/min)	33.4 ± 3.6

Mean ± Standard Deviation and %; BMI: Body Mass Index.

**Table 2:** Time-course changes in plasma lactate concentration before and after exercise SRE and SLE conditions.

Marker	Variable	Pre	0 min	30 min	60 min
Lactate (mg/dL)	SRE	13.4 ± 3.7	34.6 ± 15.9 ^##^	13.4 ± 3.7	10.3 ± 2.4
	SLE	9.9 ± 2.7	38.3 ± 16.7 ^###^	14.7 ± 4.2 ^##^	11.6 ± 3.4

Data are in mean ± Standard Deviation; SRE: Semi-Recumbent Ergometer, SLE: Side-Lying Ergometer; Pre: pre-intervention. ^##^ *p* < 0.01, ^###^ *p* < 0.001 (vs. Pre) (paired *t*-test) Using a paired *t*-test, compared to before the intervention (baseline).

**Table 3:** Time-course changes in plasma IL-6 concentration before and after exercise SRE and SLE conditions.

Marker	Variable	Pre	0 min	30 min	60 min
IL-6 (pg/mL)	SRE	0.9 ± 0.6	1.0 ± 0.6	1.3 ± 0.6 ^#^	1.1 ± 0.7
	SLE	0.7 ± 0.5	0.9 ± 0.4 ^#^	1.2 ± 0.5 ^##^	1.1 ± 0.7 ^##^

Data are in mean ± Standard Deviation; SRE: Semi-Recumbent Ergometer, SLE: Side-Lying Ergometer; Pre: pre-intervention; ^#^ *p* < 0.05, ^##^ *p* < 0.01, (vs. Pre) (paired *t*-test) Using a paired *t*-test, compared to before the intervention (baseline).

**Table 4:** Time-courses changes in plasma BDNF concentration before and after exercise SRE and SLE conditions.

Marker	Variable	Pre	0 min	30 min	60 min
BDNF (pg/dL)	SRE	30,590 ± 5879	34,910 ± 6984 ^##^	29,800 ± 6541	30,220 ± 6906
	SLE	30,880 ± 6232	34,650 ± 8393 ^#^	29,910 ± 7895	29,470 ± 5275

Data are in mean ± Standard Deviation; SRE: Semi-Recumbent Ergometer, SLE: Side-Lying Ergometer; ^#^ *p* < 0.05, ^##^ *p* < 0.01, (vs. Pre) (paired *t*-test) Using a paired *t*-test, compared to before the intervention (baseline).

## Data Availability

The original data presented in the study are openly available in Harvard Dataverse at https://doi.org/10.7910/DVN/D3TY8V, (accessed on 17 September 2025).

## Supplementary Materials

The following supporting information can be downloaded at: https://www.mdpi.com/article/10.3390/metabo15100641/s1, Table S1: Baseline characteristics of the analysis group (*n* = 10) and excluded participants (*n* = 2); Table S2: Analysis of primary outcomes showed no significant differences in lactate, IL-6, or BDNF between intervention conditions.

## List of Abbreviations

AT	Anaerobic Threshold
BDNF	Brain-Derived Neurotrophic Factor
CPX	Cardiopulmonary Exercise Testing
IL-6	Interleukin-6
SRE	Semi-Recumbent Ergometer (a novel index proposed in this study)
SLE	Side-Lying Ergometer (a novel index proposed in this study)
peak $\overset{\cdot }{V}$O_2_	Peak Oxygen Uptake

## References

[1] Pedersen B.K., Febbraio M.A. (2008). Muscle as an endocrine organ: Focus on muscle-derived interleukin-6. Physiol. Rev..

[2] Severinsen M.C.K., Pedersen B.K. (2020). Muscle-Organ Crosstalk: The Emerging Roles of Myokines. Endocr. Rev..

[3] Laurens C., Bergouignan A., Moro C. (2020). Exercise-Released Myokines in the Control of Energy Metabolism. Front. Physiol..

[4] Zunner B.E.M., Wachsmuth N.B., Eckstein M.L., Scherl L., Schierbauer J.R., Haupt S., Stumpf C., Reusch L., Moser O. (2022). Myokines and Resistance Training: A Narrative Review. Int. J. Mol. Sci..

[5] Lu Z., Wang Z., Zhang X.A., Ning K. (2024). Myokines May Be the Answer to the Beneficial Immunomodulation of Tailored Exercise-A Narrative Review. Biomolecules.

[6] Duchateau J., Stragier S., Baudry S., Carpentier A. (2021). Strength Training: In Search of Optimal Strategies to Maximize Neuromuscular Performance. Exerc. Sport. Sci. Rev..

[7] McCrory J.L., Baron H.A., Balkin S., Cavanagh P.R. (2002). Locomotion in simulated microgravity: Gravity replacement loads. Aviat. Space Environ. Med..

[8] Santos B.P., DeJong Lempke A.F., Higgins M.J., Hertel J. (2023). Influence of Reduced-Gravity Treadmill Running on Sensor-Derived Biomechanics. Sports Health.

[9] Clément G. (2017). International roadmap for artificial gravity research. npj Microgravity.

[10] Lau P., Vico L., Rittweger J. (2022). Dissociation of Bone Resorption and Formation in Spaceflight and Simulated Microgravity: Potential Role of Myokines and Osteokines?. Biomedicines.

[11] Cabral-Santos C., Castrillón C.I., Miranda R.A., Monteiro P.A., Inoue D.S., Campos E.Z., Hofmann P., Lira F.S. (2016). Inflammatory Cytokines and BDNF Response to High-Intensity Intermittent Exercise: Effect the Exercise Volume. Front. Physiol..

[12] Griffin É.W., Mullally S., Foley C., Warmington S.A., O’Mara S.M., Kelly A.M. (2011). Aerobic exercise improves hippocampal function and increases BDNF in the serum of young adult males. Physiol. Behav..

[13] Pedersen B.K., Steensberg A., Schjerling P. (2001). Exercise and interleukin-6. Curr. Opin. Hematol..

[14] Nash D., Hughes M.G., Butcher L., Aicheler R., Smith P., Cullen T., Webb R. (2023). IL-6 signaling in acute exercise and chronic training: Potential consequences for health and athletic performance. Scand. J. Med. Sci. Sports.

[15] Curovic I. (2025). The role of resistance exercise-induced local metabolic stress in mediating systemic health and functional adaptations: Could condensed training volume unlock greater benefits beyond time efficiency?. Front. Physiol..

[16] Docherty S., Harley R., McAuley J.J., Crowe L.A.N., Pedret C., Kirwan P.D., Siebert S., Millar N.L. (2022). The effect of exercise on cytokines: Implications for musculoskeletal health: A narrative review. BMC Sports Sci. Med. Rehabil..

[17] Omoto M., Matsuse H., Hashida R., Takano Y., Yamada S., Ohshima H., Tagawa Y., Shiba N. (2015). Cycling Exercise with Electrical Stimulation of Antagonist Muscles Increases Plasma Growth Hormone and IL-6. Tohoku J. Exp. Med..

[18] Abbotts K.S.S., Ewell T.R., Bomar M.C., Butterklee H.M., Bell C. (2023). Caffeine Augments the Lactate and Interleukin-6 Response to Moderate-Intensity Exercise. Med. Sci. Sports Exerc..

[19] Casado A., González-Mohíno F., González-Ravé J.M., Foster C. (2022). Training Periodization, Methods, Intensity Distribution, and Volume in Highly Trained and Elite Distance Runners: A Systematic Review. Int. J. Sports Physiol. Perform..

[20] Nilsson J., Ekblom Ö., Ekblom M., Lebedev A., Tarassova O., Moberg M., Lövdén M. (2020). Acute increases in brain-derived neurotrophic factor in plasma following physical exercise relates to subsequent learning in older adults. Sci. Rep..

[21] Müller P., Duderstadt Y., Lessmann V., Müller N.G. (2020). Lactate and BDNF: Key Mediators of Exercise Induced Neuroplasticity?. J. Clin. Med..

[22] Leal L.G., Lopes M.A., Batista M.L. (2018). Physical Exercise-Induced Myokines and Muscle-Adipose Tissue Crosstalk: A Review of Current Knowledge and the Implications for Health and Metabolic Diseases. Front. Physiol..

[23] Shero J.A., Lindholm M.E., Sandri M., Stanford K.I. (2025). Skeletal Muscle as a Mediator of Interorgan Crosstalk During Exercise: Implications for Aging and Obesity. Circ. Res..

[24] Chen Z.T., Weng Z.X., Lin J.D., Meng Z.X. (2024). Myokines: Metabolic regulation in obesity and type 2 diabetes. Life Metab..

[25] Son B.K., Nanao-Hamai M., Umeda-Kameyama Y., Lyu W., Tanaka T., Yoshizawa Y., Akishita M., Iijima K. (2025). Ikigai is associated with lower incidence of frailty during a 5-year follow-up in older women: The possible role of interleukin-6. Arch. Gerontol. Geriatr..

[26] Phillips C. (2017). Brain-Derived Neurotrophic Factor, Depression, and Physical Activity: Making the Neuroplastic Connection. Neural Plast..

[27] Biolo G., Ciocchi B., Stulle M., Bosutti A., Barazzoni R., Zanetti M., Antonione R., Lebenstedt M., Platen P., Heer M. (2007). Calorie restriction accelerates the catabolism of lean body mass during 2 wk of bed rest. Am. J. Clin. Nutr..

[28] Juhl O.J., Buettmann E.G., Friedman M.A., DeNapoli R.C., Hoppock G.A., Donahue H.J. (2021). Update on the effects of microgravity on the musculoskeletal system. npj Microgravity.

