# Supplementary material for: Matched Metabolic Stress Preserves Myokine Responses Regardless of Mechanical Load: A Randomized, Controlled Crossover Trial
Source: Metabolites. 2025 Sep 25;15(10):641. doi: 10.3390/metabo15100641 (PMC12566153; doi:10.3390/metabo15100641)
Supplement: Supplementary file 1 [file metabolites-15-00641-s001.zip › Supplementary Table S1.pdf]

Supplementary Table S1. Baseline characteristics of the analysis group (n = 10) and excluded participants (n = 2)

**Variable**	**Analysis Group (n=10, Mean ± SD)**	**Excluded ID1**	**Excluded ID2**	**Excluded Mean (n=2, Mean ± SD)**	**SMD**
Age (years)	21.2±0.9	23	21	22.0±1.4	-0.83
Length (cm)	173.8±5.9	173	179	176±4.2	-0.38
Weight (kg)	64.9±8.3	64	60	62±2.8	0.37
BMI (kg/m^2)	21.3 ± 2.0	21.3	18.7	20.0 ± 1.8	0.66
PeakVO2(mL/kg/min)	33.2±3.9	34.4	35.2	34.8±0.5	-0.43

Baseline characteristics of the analysis group (n=10) and the excluded group (n=2). Data are presented as mean ± standard deviation (SD). Standardized mean differences (SMD) were calculated to evaluate the magnitude of differences between groups. An SMD of 0.2, 0.5, and 0.8 are typically interpreted as small, medium, and large differences, respectively. Age showed a large difference (SMD = 0.83), while BMI indicated a moderate difference (SMD = 0.66). Other variables, including length, weight, and peak VO_2, showed only small differences between the groups.
