# Supplementary material for: Matched Metabolic Stress Preserves Myokine Responses Regardless of Mechanical Load: A Randomized, Controlled Crossover Trial
Source: Metabolites. 2025 Sep 25;15(10):641. doi: 10.3390/metabo15100641 (PMC12566153; doi:10.3390/metabo15100641)
Supplement: Supplementary file 1 [file metabolites-15-00641-s001.zip › Supplementary Table S2.pdf]

Factor	BDNF Estimate (p-value) [95% CI]	IL-6 Estimate (p-value) [95% CI]	Lactate Estimate (p-value) [95% CI]
Sequence	-1931.114(p=0.2081) [-5178.216, 1315.988]	-0.1222(p=0.4771) [-0.4980, 0.2536]	17.2202(p=0.0242) [2.8727, 31.5677]
Period	5123.861(p=0.1329) [-1770.262, 12017.994]	0.4363(p=0.2180) [-0.2931, 1.1656]	36.3414(p=0.0446) [1.0041, 71.6788]
Group (Exercise intervention)	1410.523(p=0.2686) [-1325.638, 4146.742]	0.0195(p=0.8757) [-0.2588, 0.2971]	-3.7298(p=0.5993) [-19.3568, 11.8972]
Carryover	-6284.418(p=0.0884) [-13638.98, 1070.140]	-0.6558(p=0.0931) [-1.4371, 0.1256]	-35.7162(p=0.0582) [-72.9393, 1.4161]

Supplementary Table S2

Analysis of primary outcomes showed no significant differences in lactate, IL-6, or BDNF between intervention conditions. However, order effects and period effects reached statistical significance for lactate.
